# Editorial: Nonlinearity in Living Systems: Theoretical and Practical Perspectives on Metrics of Physiological Signal Complexity

**DOI:** 10.3389/fphys.2019.00298

**Published:** 2019-03-29

**Authors:** Sladjana Z. Spasić, Srdjan Kesić

**Affiliations:** ^1^Department of Life Sciences, Institute for Multidisciplinary Research, University of Belgrade, Belgrade, Serbia; ^2^Faculty of Informatics and Computing, Singidunum University, Belgrade, Serbia; ^3^Department of Neurophysiology, Institute for Biological Research “Siniša Stanković”, University of Belgrade, Belgrade, Serbia

**Keywords:** fractal physiology, non-linearity, electroencephalograph, hearth beat, fractal dimension, non-linear measures, disease

Despite the extraordinary development of physiology in recent decades, we still struggle to understand the multilevel organization and functional integration in complex biological systems (Goldberger et al., 2002; West, 2010). In this respect, the use of non-linear descriptors and models in physiology is becoming an essential part of current and future efforts to understand complex biological systems in both health and disease (Goldberger et al., 2002). Nonetheless, non-linear-based methods have been utilized in solving many fascinating problems in different subfields of physiology and pathophysiology.

In this Research Topic, we present original research articles, reviews and new hypotheses that extend our knowledge and understanding of fractal physiology and its interdisciplinary nature. Many of physiological researches in recent decades has focused on quantitative analysis, characterization of dynamics across time scales, and ultimately control of functional physiological networks, especially in the central and peripheral nervous system (West, 2010; França et al., 2018). Also, one of the greatest challenges of fractal physiology is to elucidate the fractal and non-linear mechanisms involved in the generation and allometric control of complex physiological networks, for which we know that they are a product of several different and interacting temporal scales (Goldberger et al., 2002; West, 2010; França et al. 2018). Therefore, it would not be wrong to emphasize that the future of physiology lies in finding ways for applying existing non-linear measures, developing new ones, and setting new hypotheses aimed at understanding the variability of physiological time series in health and disease. In this respect, Bohara et al. provide a new hypothesis with valuable theoretical insight into the relationship between crucial events (1/f noise) and the wave-like nature of the brain processes. The results of this paper confirm an important role of crucial events in the dynamics of the brain; they provide theoretical tools necessary to understand the joint action of crucial events and periodicity, which can be helpful in better understanding of cognitive processes. von Wegner proposed an information-theoretic approach to numerically determine the Markov order of discrete processes defined over a finite space. This approach uses a combination of autoinformation and partial autoinformation to deal with Markov and non-Markov processes with known stochastic properties and can be used for discrete transformation of electroencephalograph (EEG) data sets as well.

Many of the contributions highlight the non-linear behavior of various components of an organism (“vertical” component of complexity) from the cellular to tissue and organ levels work together to maintain complex physiological networks. Among the multiplicity of non-linear measures, Higuchi fractal dimension (HFD), used alone or in combination with other measures, once again proves to be a valuable and important source of new information about neuronal networks underlying time scale invariant EEG dynamics. In that regard, Croce et al. used HFD and detrended fluctuation analysis (DFA) to investigate circadian rhythms in fractal features of EEG signals. These authors showed that HFD can be useful for monitoring circadian fluctuations of fractal features of EEG at rest in both eyes closed and eyes open conditions. Päeske et al. employed a set of non-linear measures such as HFD, Katz fractal dimension (KFD), Lempel-Ziv complexity (LZC), sample entropy (SampEn), and synchronization likelihood (SL) to estimate the impact of the strong cyclic signal component on the result of surrogate data method in the case of resting EEG signals (80 healthy human subjects) and the impact of segment length on this method. The authors have found that in the case of the signals with non-monotonic spectrum and strong dominant frequency, the correct use of surrogate data method requires the signal length comprising of full periods of the spectrum dominant frequency. This study once more highlights the importance of correct selection of EEG signal segment length for the surrogate data method to estimate non-linearity. Lebiecka et al. showed that HFD may be a useful marker for evaluation of the repetitive transcranial magnetic stimulation (rTMS) effectiveness and the therapy progress as well as for group differentiation between major depression disorder (MDD) and bipolar disorder (BP), or between responders and non-responders. In this research topic, the significance of HFD as a non-linear measure for different time series including electrocardiograph (ECG) signals has been confirmed. For instance, Gomolka et al. performed HFD analysis of heart rate variability (HRV) in order to assess the autonomic nervous system (ANS) sympathetic and parasympathetic activity in healthy and diabetic individuals. They concluded that HFD may be used for assessment of percutaneous auricular vagus nerve stimulation (pVNS) on ANS, to provide stimulation feedback for online regulation of therapy in a fast and robust way. Furthermore,  Ahammer et al. simultaneously used video recordings and electrode registration of potentials to characterize beat to beat variability of cardiac tissue in control conditions and during acetylcholine stimulation. By using variation analyses of video recordings with two distinguished non-linear measures, SampEn and HFD, these authors showed that high-speed video camera technique might represent a non-invasive tool that allows long-lasting recordings for detecting variations in beating dynamics during varying conditions.

The use of other non-linear measures and their specific combinations also occupy a significant place in this research topic. Thus, Liang et al. employed DFA combined with surrogate data method to measure the long-range temporal correlations (LRTCs) of the EEG signals of patients with the minimal conscious state after the spinal cord stimulation (SCS). Authors concluded that the brain activities at low-frequency oscillations, particularly in the frontal and occipital regions, were improved by SCS. By using cross recurrence quantification analysis (cRQA) of the coupling of the gaze and postural sway to the motion of a visual stimulus, Haworth and Stergiou showed that chaos is an invariant and beneficial feature of biological motion, a feature which may be critical for immediate and robust coordination of the self with the environment and other environmental agents. In a similar manner, Andreo et al. applied Box-counting FD as well Minkowski-Bouligand FD measure of dynamic changes in the center of mass during a set movement that indicated real-time processing effects during a balance task associated with the type of taping used to enhance postural stability. Hwang et al. applied stabilogram diffusion analysis and showed that amplification of low-frequency errors improves force control by shifting relative significance of feedforward and feedback processes. Lucas et al. combined Shannon entropy, wavelets, mean bending energy in order to devise automated determination of foot type. Their research suggests that automated wavelet-based foot type classification of 2D binary images of the plantar surface of the foot is comparable to current state-of-the-art methods providing a cost and time effective tool suitable for clinical diagnostics. In their study Solé-Casals at al. proposed a methodology for extracting of handwriting features using the discrete Fourier transform (DFT) and derive an algorithm in order to discriminate between control and subjects with essential tremor (ET). Authors showed that the radius and residues of XY positions of an Archimedes' spiral, could be used to detect ET with a performance of almost 85 and 96%. The results indicated that it is possible to detect ET from controls using a reduced set of features, which makes this method feasible for implementation in the portable form of the device and on the web network. Ibáñez-Molina et al. used Lempel-Ziv complexity (LZC) and Multiscale LZC to reveal EEG multiscale complexity in schizophrenia patients during picture naming. In addition to showing that patients and controls showed a different pattern of brain complexity depending on their cognitive state (at rest or under cognitive challenge), these authors once more demonstrated that non-linear approaches to EEG signal analysis can help to characterize brain dysfunction in schizophrenia.

Two original papers dealt with the correlation between the neurodegenerative process underlying cognitive impairment and retinal vascular complexity. Cabrera DeBuc et al. used information dimension, correlation dimension, FD measured by box counting method for quantification the correlation between the retinal vascular complexity and neurodegenerative changes in patients with cognitive impairment (CI). This paper showed that there are multimodal retinal markers that may be sensitive to CI decline, suggesting that retinal geometric vascular and functional parameters might be associated with physiological changes in the retina due to CI. In a similar manner, Kostic et al. illustrated that the FD of the foveal vessel arborization could provide useful information to identify early morphological changes in the retina of patients with type 2 diabetes mellitus. Despite the significant progress in the development and application of non-linear methodology in biomedical research, there is still room for the use of linear methods in the analysis of electrophysiological signals. In that respect, Jalilifar et al. have evaluated antiepileptogenic effects of low-frequency stimulation (LFS) before or after kindling using spectral power analysis (theta/alpha ratio) of EEG signals.

The development of automated systems that can predict the course of the disease is one of the greatest challenges of modern pathophysiology that can provide better outcomes of medical interventions. In recent times, there is an increasing interest in adaptive neuro-fuzzy inference systems (ANFIS) and their application to non-linear dynamical systems in health and disease. Accordingly, Yadollahpour et al. have developed ANFIS based medical decision support system to predict chronic kidney disease progression.

The “horizontal” component of complexity is also not neglected in this topic. In that regard, Eguiraun et al. proposed a non-linear analysis and Shannon entropy to characterize the relationship of the constituted complex entities with other complex entities (interactions between individuals of *European seabass*). Finally, the topic includes the review of measures and metrics of biological systems. Kasum et al. summarize and reviews different mathematical and informational approaches to biological systems, providing an interesting overview of the complex relationship between experimental biomedical research on one hand and computational science and mathematics on other.

Although a common thread runs between them, each article in this research topic proposes distinctive applications of non-linear methodology in physiology and deserves attention in its own right. Together, they illustrate the importance and vitality of fractal research in physiology and medicine. Whether through an effort to improve our understanding of crucial events as an important process of self-organization; consideration of how best to exploit and combine non-linear measures; extension of applications of these measures to new physiological and pathological conditions, the authors here develop engaging proposals for investigating “horizontal” and “vertical” components of complexity in biological systems.

## Author Contributions

All authors listed have made a substantial, direct and intellectual contribution to the work, and approved it for publication.

### Conflict of Interest Statement

The authors declare that the research was conducted in the absence of any commercial or financial relationships that could be construed as a potential conflict of interest.
